# Mr. Gladstone and the Medical Profession

**Published:** 1892-07-23

**Authors:** 


### The Hospital

An Institutional Journal of

**Science, Medicine, Nursing, and Philanthropy.**

*For Hospitals, Asylums, Infirmaries, Dispensaries, Medical Practitioners, Students, Nurses, and the Charitable Public.*

Vol. XII.—No. 304.] [July 23, 1892.

### Mr. Gladstone and the Medical Profession.

The whole attention of the country has been fixed on the General Election during the last few weeks, and we deal with one phase of it, election fever, in another column. Of course, politics are altogether outside and apart from medical journalism, and with them we have nothing to do. Still, politics as a profession attract the most ambitious, and also some of the best specimens of the race, and so politicians excite and fix attention more than any other class. There is one figure, however, which stands out prominently from the rest as a wonderful example of the staying power vested in a sound constitution, which may successfully resist the ravages of time, providing a man's system be properly husbanded, and especially providing it be protected from enervating tendencies and indiscretions as to diet, exercise, and other simple but necessary matters, to the non-observance of which premature decay is largely due.

It must be interesting to inquire how it is that Mr. Gladstone, although eighty-two years of age, has shown himself capable of leading a political contest in which he has taken the most active part, without injury to his health, and apparently without experiencing great, or even inconvenient fatigue. From inquiries we have made, we believe Mr. Gladstone's astonishing robustness to be due o three causes: A sound constitution, which he has had the wisdom to husband and protect; a devoted wife, who has allowed nothing to prevent her from giving the closest continuous attention to the well-being, preservation, and protection from worry of her husband; and the fact that, thanks to a grateful patient of the London Hospital, Mr. Gladstone's health has been committed to the medical charge of a physician, Sir Andrew Clark, than whom no one is better qualified, by high scientific attainment and sound common sense, to shield a man of affairs from the many risks which have in past times frequently cut short the career of eminent statesmen. A man is, after all, very frequently his own best physician, although it has been said with truth, that self doctors have fools for their patients. Still, no medical care can equal the precautionary measures a man can himself take where his own health is concerned. All of us need every day not too much, but at the same time enough good food, and an adequate time in bed and asleep, sleep being the rock on which many men go to pieces in these days of hurry and rush. Now, to be certain of securing sleep, it is essential to take each day a sufficiency of outdoor exercise so that the system may become physically tired. Hence, Mr. Gladstone's love of tree felling has probably done more than anything else to conserve his wonderful staying power. In such matters as diet and sleep, and any one normally sound in health, who attends to them, is nearly sure to be free from serious illness, each of us ought to be capable of adequately providing for himself. In the case of ordinary mortals a few simple precautions will secure, that the individual shall live, and not merely exist, seeing that life is not to live, but to be well. All this Mr. Gladstone has discovered and learnt to believe, and so he has come to be to-day a phenomenal instance of the best physical type of our race. The family of Mr. Gladstone have devoted themselves to their father's interests for years. Everything is so regulated as to secure to Mr. Gladstone the minimum of worry, and he is saved from all avoidable fatigue. If work is wanted to be done, there are always willing hands free to undertake and complete it, without delay or remonstrance. All correspondence is carefully sorted and weighed, and none but important letters are brought to notice on busy days. Mrs. Gladstone's solicitude for and care of her husband is a notable example for all wives, and to her care Mr. Gladstone has often attributed his escapes from illness, and not a little of his continuous good health. Yet Mrs. Gladstone never neglects her other duties. We can, speaking from personal experience declare, that Mrs. Gladstone at the very busiest time of the year, has most cheerfully and effectually aided the hospitals by her pen, whilst her convalescent home at Woodford is one of the most economically-conducted institutions in the world.

The Premier of the British Empire has little time to think of himself or his own health and requirements. The strain upon mind and body is truly appalling to those who have a precise knowledge of the duties and responsibilities of this high office of State. So the duty of conserving and maintaining the health of the head of Her Majesty's Government is by no means a light one, but Mr. Gladstone's condition to-day proves that Sir Andrew Clark is *facile princeps.* The physician in charge of such a patient has to look after the regime, diet, exercise, and hours of rest, to see that there is no undue strain in any one direction, and to exercise sufficient tact to win his patient to like the ways, which in his case tend to the continuance of sound health. All this and much more Sir Andrew has successfully accomplished in Mr. Gladstone's case, and the credit of this remarkable success must be shared by Mrs. Gladstone, to whom Sir Andrew would be the first to acknowledge his indebtedness. The medical profession and all wives who love their husbands may carefully ponder the facts we have been considering, for they contain a mine of practical wealth for those who can learn as they read and ponder.

It is now certain that Mr. Gladstone will once again become Prime Minister of England and head of Her Majesty's Government. He owes, under Providence, that position to-day in no small degree to the causes we have been considering. One of these causes the services which medicine renders to the State and to all its leaders and governors we commend to Mr. Gladstone's most generous and earnest consideration. It is a great honour to be Prime Minister, but it is even a greater distinction to be Prime Minister at eighty-two years of age, probably the first example of the kind in history. If medical science has had ought to do with Mr. Gladstone's position to-day, and who can doubt that it has had much, what higher duty can devolve upon him as Prime Minister, than to mark his assumption of office by advising the Queen to at last do justice to medicine, by creating at least two medical peers. Sir William Jenner and Sir James Paget, a physician and a surgeon respectively of world-wide reputation, have personally well deserved the honour, and the medical profession would be ennobled by their selection for it. This is one of those social reforms which press for immediate solution. Is Jenner or Paget less worthy of a life peerage than the eminent men who now sit on the bench of Bishops, or than any of the lawyers, soldiers, or sailors, who have been rewarded by hereditary peerages? Can any member of the House of Lords do greater service to his country in that assembly than an eminent member of the medical profession could render in the promotion of legislation for securing and protecting the public health? Yet, despite the enormous saving of life effected under Providence by the direct labours of many eminent doctors, the Government of this country has never adequately recognised the fact. Let it be Mr. Gladstone's privilege and pleasure to put this matter right at once. Let his be the hand to show, that the time has gone by in this country, when honours and rewards are distributed amongst those who destroy life, whilst those who save it are neglected and passed by. Such an act would redound to the lasting honour of our octogenarian Premier, who has more than once expressed his personal indebtedness to medicine and her votaries. We shall, indeed, be surprised if this great, this unique occasion is allowed to pass without such a lasting record of its existence and importance to the State.

